# Complete genome sequence of *Thermobispora bispora* type strain (R51^T^)

**DOI:** 10.4056/sigs.962171

**Published:** 2010-06-15

**Authors:** Konstantinos Liolios, Johannes Sikorski, Marlen Jando, Alla Lapidus, Alex Copeland, Tijana Glavina, Del Rio, Matt Nolan, Susan Lucas, Hope Tice, Jan-Fang Cheng, Cliff Han, Tanja Woyke, Lynne Goodwin, Sam Pitluck, Natalia Ivanova, Konstantinos Mavromatis, Natalia Mikhailova, Olga Chertkov, Cheryl Kuske, Amy Chen, Krishna Palaniappan, Miriam Land, Loren Hauser, Yun-Juan Chang, Cynthia D. Jeffries, John C. Detter, Thomas Brettin, Manfred Rohde, Markus Göker, James Bristow, Jonathan A. Eisen, Victor Markowitz, Philip Hugenholtz, Hans-Peter Klenk, Nikos C. Kyrpides

**Affiliations:** 1 DOE Joint Genome Institute, Walnut Creek, California, USA; 2 DSMZ – German Collection of Microorganisms and Cell Cultures GmbH, Braunschweig, Germany; 3 Los Alamos National Laboratory, Bioscience Division, Los Alamos, New Mexico, USA; 4 Biological Data Management and Technology Center, Lawrence Berkeley National Laboratory, Berkeley, California, USA; 5 Oak Ridge National Laboratory, Oak Ridge, Tennessee, USA; 6 HZI – Helmholtz Centre for Infection Research, Braunschweig, Germany; 7 University of California Davis Genome Center, Davis, California, USA

**Keywords:** Two distinct 16S rRNA genes, strictly thermophilic, non-pathogenic, *Streptosporangineae*, GEBA

## Abstract

*Thermobispora bispora* (Henssen 1957) Wang *et al.* 1996 is the type species of the genus *Thermobispora*. This genus is of great interest because it is strictly thermophilic and because it has been shown for several of its members that the genome contains substantially distinct (6.4% sequence difference) and transcriptionally active 16S rRNA genes. Here we describe the features of this organism, together with the complete genome sequence and annotation. This is the second completed genome sequence of a member from the suborder *Streptosporangineae* and the first genome sequence of a member of the genus *Thermobispora*. The 4,189,976 bp long genome with its 3,596 protein-coding and 63 RNA genes is part of the *** G****enomic* *** E****ncyclopedia of* *** B****acteria and* *** A****rchaea * project.

## Introduction

**Figure 1:**
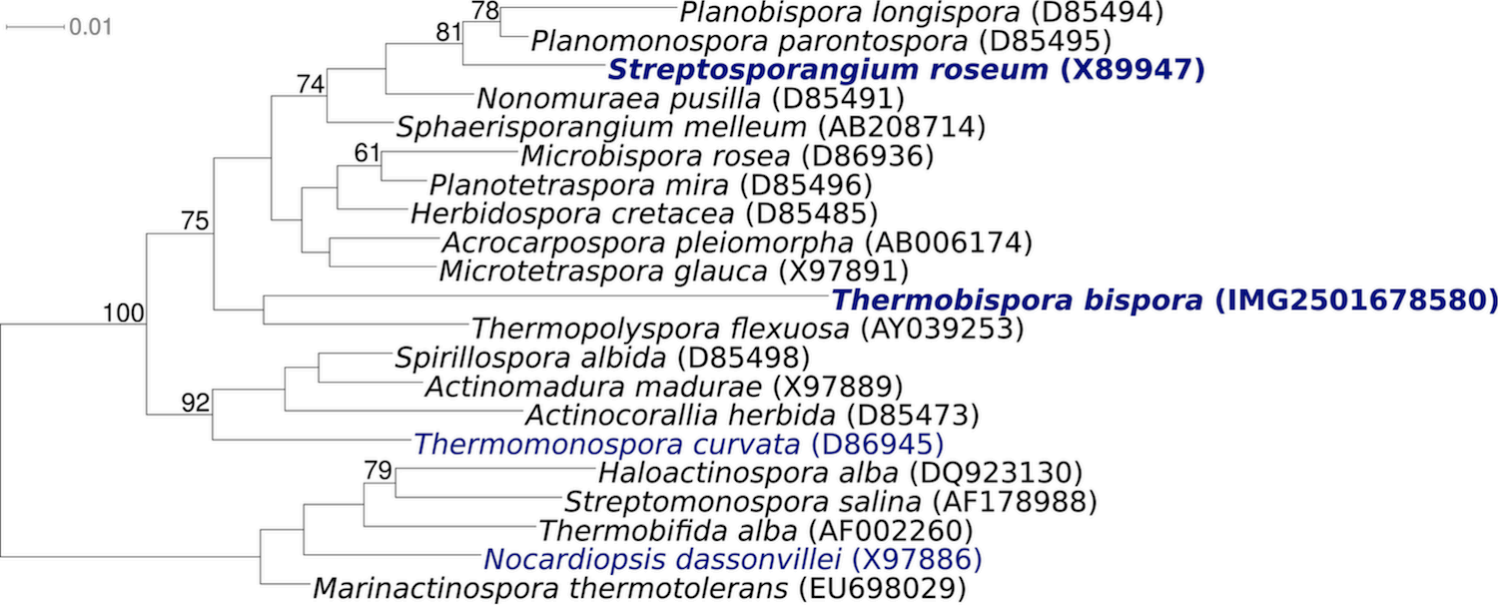
Phylogenetic tree highlighting the position of *T. bispora* R51^T^ relative to the type strains of the other genera within the suborder *Streptosporangineae* (except for *Actinoallomurus*, which was published after the analysis was completed). The tree was inferred from 1,371 aligned characters [15,16] of the 16S rRNA gene sequence under the maximum likelihood criterion [17] and rooted in accordance with the current taxonomy [18]. The branches are scaled in terms of the expected number of substitutions per site. Numbers above branches are support values from 1,000 bootstrap replicates if larger than 60%. Lineages with type strain genome sequencing projects registered in GOLD [19] are shown in blue, published genomes in bold, *e.g*. the recently published GEBA genome from *Streptosporangium roseum* [20].

Strain R51^T^ (= DSM 43833 = ATCC 19993 = JCM 10125) is the type strain of the species *Thermobispora bispora*, which is the type species of the genus *Thermobispora* [1]. The generic name of the genus derives from the Greek words ‘thermos’, ‘bis’, and ‘spora’, to indicate high temperature two-spored organisms [1]. Strain R51^T^ was isolated from decaying manure in Berlin (Germany) in 1954 [2]. Other strains were isolated during the same research project from other types of manure in other cities in Germany and in Finland [2]. As deduced from 16S gene sequences, *T. bispora* was also found in compost in Sweden [3]. Historically, strain R51^T^ was originally classified in 1957 as *Thermopolyspora bispora* [2]. At the same time, a morphologically similar genus, *Microbispora,* was described [4], which has priority and *T. bispora* was subsequently transferred to the genus *Microbispora* [5,6]. However, based on thermal preferences [2,7], chemotaxonomic features [7], and the two-dimensional polyacrylamide gel electrophoresis patterns of the ribosomal protein AT-L30 [8], *Microbispora bispora* was subsequently removed from the genus *Microbispora* to be the type species of the new genus *Thermobispora* [1]. *T. bispora* is currently the only species in the genus *Thermobispora* [1]. In 1997 *T. bispora* gained interest, as it was described as the first organism to have two distinct (6.4% of total nucleotides) types of transcriptionally active 16S rRNA genes (GenBank accessions U83909 and U83912) [9]. Based on the two copies of the 16S rRNA genes that match best to sequence U83909 the closest related type strain (9% sequence difference [10]) is *Micromonospora pattaloongensis* [11] of the family *Micromonosporaceae*; based on the two copies of the 16S rRNA genes that match best to sequence U83912 the closest related type strain (8% sequence difference [10]) is *Planotetraspora silvatica* [12] of the family *Streptosporangiaceae*. Neither fit to the taxonomic position as shown in the List of Procaryotic names with Standing in Nomenclature that shows the genus *Thermobispora* as a member of the family *Pseudonocardiaceae*, reflecting the current uncertainty of the taxonomic position of *T. bispora*  [13]. In their recent review of *Actinobacteria* taxonomy, Zhi *et al.* [14] suggested to place *Thermobispora* in the suborder *Streptosporangineae* without assignment to a family, which is in accordance with our SSU rRNA tree (Figure 1). 16S rRNA sequences from environmental samples and metagenomic surveys with both 16S rRNA sequences detected phylotypes with approximately 89-92% 16S rRNA gene sequence similarity to both (U83909 and U83912) reference sequences only in a compost metagenome [21], indicating a very rare occurrence of *Thermobispora*-spp. in the environment (status March 2010). Here we present a summary classification and a set of features for *T. bispora* R51^T^, together with the description of the complete genomic sequencing and annotation.

## Classification and features

Figure 1 shows the phylogenetic neighborhood of for *T. bispora* R51^T^ in a 16S rRNA based tree. The sequences of the four 16S rRNA gene copies in the genome differ from each other by up to 94 nucleotides, and differ by up to 95 nucleotides from the previously published 16S rRNA sequence generated from ATCC 19993 (U58523).

**Figure 2:**
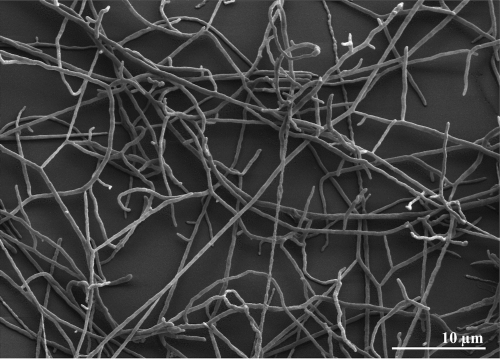
Scanning electron micrograph of *T. bispora* R51^T^

*T. bispora* cells form substrate mycelia whose hyphae are 0.5 to 0.8 µm in diameter [1] (Figure 2). The aerial mycelia branch monopodally and bear longitudinal pairs of spores [1] (not visible in Figure 2). The spore diameters are usually 1.2 to 2.0 µm, but in liquid media spores with a diameter of 3 µm may occur [1]. The aerial mycelia are white, and the substrate mycelia are yellow or yellowish brown on the media used in the respective study (International *Streptomyces* Project medium 4 agar and IF0328 agar; Institute for Fermentation) [1]. No soluble pigment is produced [1]. *T. bispora* is an obligately thermophilic organism (Table 1) [1]. Starch is not hydrolyzed; inositol and rhamnose are utilized for growth, but arabinose and glycerol are not utilized [1]. Also, *T. bispora* is negative for iodinin production and nitrate reduction [1].

**Table 1:** Classification and general features of *T. bispora* R51^T^ according to the MIGS recommendations [22]

**MIGS ID**	**Property**	**Term**	**Evidence code**
	Current classification	Domain *Bacteria*	TAS [23]
		Phylum ‘*Actinobacteria*’	TAS [13]
		Class *Actinobacteria*	TAS [24]
		Subclass *Actinobacteridae*	TAS [14,24]
		Order *Actinomycetales*	TAS [14]
		Suborder *Streptosporangineae*	TAS [14]
		Family Incertae sedis	TAS [14]
		Genus *Thermobispora*	TAS [1]
		Species *Thermobispora bispora*	TAS [2]
		Type strain R51	TAS [5]
	Gram stain	positive	TAS [1]
	Cell shape	mycelia with hyphae	TAS [2]
	Motility	non-motile	TAS [1]
	Sporulation	sporulating	TAS [1]
	Temperature range	thermophile, 50°C - 65°C	TAS [1]
	Optimum temperature	not determined	TAS [1]
	Salinity	not determined	TAS [1]
MIGS-22	Oxygen requirement	aerobic	TAS [1,2]
	Carbon source	inositol and rhamnose	TAS [1]
	Energy source	sugars	TAS [1]
MIGS-6	Habitat	compost and other decaying material	TAS [2,3]
MIGS-15	Biotic relationship	unknown	
MIGS-14	Pathogenicity	not reported	
	Biosafety level	1	TAS [25]
	Isolation	decaying mixed manure	TAS [2]
MIGS-4	Geographic location	Berlin, Germany	TAS [2]
MIGS-5	Sample collection time	September 30, 1954	TAS [2]
MIGS-4.1 MIGS-4.2	Latitude Longitude	52.52 14.42	NAS
MIGS-4.3	Depth	not reported	
MIGS-4.4	Altitude	not reported	

Evidence codes - IDA: Inferred from Direct Assay (first time in publication); TAS: Traceable Author Statement (i.e., a direct report exists in the literature); NAS: Non-traceable Author Statement (i.e., not directly observed for the living, isolated sample, but based on a generally accepted property for the species, or anecdotal evidence). These evidence codes are from of the Gene Ontology project [26]. If the evidence code is IDA, then the property was directly observed for a live isolate by one of the authors or an expert mentioned in the acknowledgements.

### Chemotaxonomy

The cell wall of strain R51^T^ contains predominantly the menaquinone MK-9(H_0_) (75%) and only small amounts of MK-9(H_2_) and MK-9(H_4_) [7]. Strain R51^T^ has a type PIV phospholipid pattern, and contains phosphatidylethanolamine but not phosphatidylglycerol and trace amounts of glucosamine-containing phospholipids [7]. The cell wall contains a major amount of *meso*-diaminopimelic acid, and the whole-cell hydrolysate contains madurose and galactose [1]. The fatty acid composition of strain R51^T^ is dominated by saturated acids, with iso-C_16:0_ (55%) being the most frequent acid, followed by anteiso-C_17:0_ (8%), the unsaturated C_18:1_ (8%), iso-C_18:0_ (6%) and C_16:0_ [7]. Also, strain R51^T^ contains minor amounts of 10-methyl-branched chain fatty acids [7].

## Genome sequencing and annotation

### Genome project history

This organism was selected for sequencing on the basis of its phylogenetic position [27], and is part of the *** G****enomic* *** E****ncyclopedia of* *** B****acteria and* *** A****rchaea * project [28]. The genome project is deposited in the Genome OnLine Database [19] and the complete genome sequence is deposited in GenBank. Sequencing, finishing and annotation were performed by the DOE Joint Genome Institute (JGI). A summary of the project information is shown in Table 2.

**Table 2:** Genome sequencing project information

**MIGS ID**	**Property**	**Term**
MIGS-31	Finishing quality	Finished
MIGS-28	Libraries used	Three genomic libraries: two Sanger libraries – 8 kb pMCL200 and fosmid pcc1FOS and one 454 pyrosequece standard library
MIGS-29	Sequencing platforms	ABI3730, 454 GS FLX
MIGS-31.2	Sequencing coverage	7.1× Sanger; 1.1× pyrosequence pseudo-reads
MIGS-30	Assemblers	Newbler version 1.1.02.15, phrap
MIGS-32	Gene calling method	Prodigal, GenePRIMP
	INSDC ID	CP001874
	Genbank Date of Release	May 17, 2010
	GOLD ID	Gc01281
	NCBI project ID	469371
	Database: IMG-GEBA	2501651196
MIGS-13	Source material identifier	DSM 43833
	Project relevance	Tree of Life, GEBA

### Growth conditions and DNA isolation

*T. bispora* strain R51^T^, DSM 43833, was grown in DSMZ medium 84 (Rolled oats mineral medium) [29] at 55°C. DNA was isolated from 1-1.5 g of cell paste using Qiagen Genomic 500 DNA Kit (Qiagen, Hilden, Germany) with lysis modification st/FT according to Wu *et al*. [28].

### Genome sequencing and assembly

The genome of *T. bispora* was sequenced using a combination of Sanger and 454 sequencing platforms. All general aspects of library construction and sequencing can be found at http://www.jgi.doe.gov/. 454 pyrosequencing reads were assembled using the Newbler assembler version 1.1.02.15 (Roche). Large Newbler contigs were broken into 4,798 overlapping fragments of 1,000 bp and entered into assembly as pseudo-reads. The sequences were assigned quality scores based on Newbler consensus q-scores with modifications to account for overlap redundancy and to adjust inflated q-scores. A hybrid 454/Sanger assembly was made using the parallel phrap assembler (High Performance Software, LLC). Possible mis-assemblies were corrected with Dupfinisher or transposon bombing of bridging clones [30]. Gaps between contigs were closed by editing in Consed, custom primer walk or PCR amplification. A total of 1,181 Sanger finishing reads were produced to close gaps, to resolve repetitive regions, and to raise the quality of the finished sequence. The error rate of the completed genome sequence is less than 1 in 100,000. The final assembly consists of 40,290 Sanger and 1.1× pyrosequence based pseudo-reads. Together Sanger reads and pseudo-reads provided 8.19× coverage of the genome.

### Genome annotation

Genes were identified using Prodigal [31] as part of the Oak Ridge National Laboratory genome an-notation pipeline, followed by a round of manual curation using the JGI GenePRIMP pipeline [32]. The predicted CDSs were translated and used to search the National Center for Biotechnology Information (NCBI) nonredundant database, UniProt, TIGRFam, Pfam, PRIAM, KEGG, COG, and InterPro databases. Additional gene prediction analysis and manual functional annotation was performed within the Integrated Microbial Genomes Expert Review (IMG-ER) platform [33].

## Genome properties

**Figure 3:**
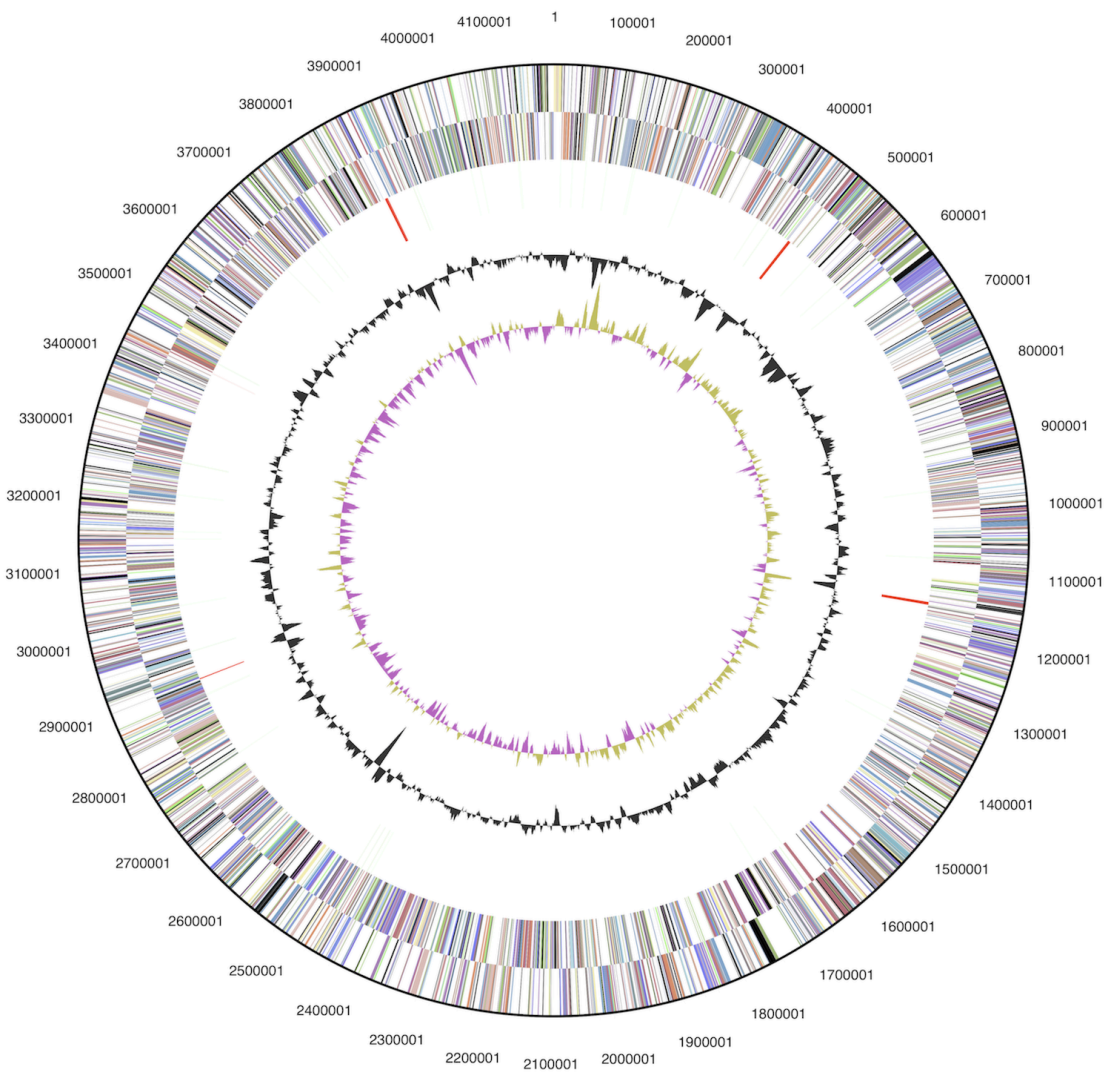
Graphical circular map of the genome. From outside to the center: Genes on forward strand (color by COG categories), Genes on reverse strand (color by COG categories), RNA genes (tRNAs green, rRNAs red, other RNAs black), GC content, GC skew.

The genome is 4,189,976 bp long and comprises one main circular chromosome with an overall GC content of 72.4% (Table 3 and Figure 3). Of the 3,659 genes predicted, 3,596 were protein-coding genes, and 63 RNAs; fifty pseudogenes were also identified. The majority of the protein-coding genes (71.9%) were assigned with a putative function while the remaining ones were annotated as hypothetical proteins. The distribution of genes into COGs functional categories is presented in Table 4.

**Table 3:** Genome Statistics

**Attribute**	Value	% of Total
Genome size (bp)	4,189,976	100.00%
DNA coding region (bp)	3,548,135	84.68%
DNA G+C content (bp)	3,034,765	72.43%
Number of replicons	1	
Extrachromosomal elements	0	
Total genes	3,659	100.00%
RNA genes	63	1.72%
rRNA operons	3	
Protein-coding genes	3,596	98.28%
Pseudo genes	50	1.37%
Genes with function prediction	2,632	71.93%
Genes in paralog clusters	491	13.42%
Genes assigned to COGs	2,610	71.33%
Genes assigned Pfam domains	2,844	77.73%
Genes with signal peptides	795	21.73%
Genes with transmembrane helices	864	23.61%
CRISPR repeats	6	

**Table 4:** Number of genes associated with the general COG functional categories

**Code**	**value**	**%age**	**Description**
J	149	5.0	Translation, ribosomal structure and biogenesis
A	1	0.0	RNA processing and modification
K	304	10.3	Transcription
L	141	4.8	Replication, recombination and repair
B	1	0.0	Chromatin structure and dynamics
D	21	1.0	Cell cycle control, cell division, chromosome partitioning
Y	0	0.0	Nuclear structure
V	48	1.6	Defense mechanisms
T	192	6.5	Signal transduction mechanisms
M	140	4.7	Cell wall/membrane biogenesis
N	3	0.1	Cell motility
Z	0	0.0	Cytoskeleton
W	0	0.0	Extracellular structures
U	29	1.0	Intracellular trafficking, secretion, and vesicular transport
O	98	3.3	Posttranslational modification, protein turnover, chaperones
C	204	6.9	Energy production and conversion
G	221	7.5	Carbohydrate transport and metabolism
E	279	9.4	Amino acid transport and metabolism
F	82	2.8	Nucleotide transport and metabolism
H	146	4.9	Coenzyme transport and metabolism
I	133	4.5	Lipid transport and metabolism
P	138	4.7	Inorganic ion transport and metabolism
Q	85	2.9	Secondary metabolites biosynthesis, transport and catabolism
R	351	11.9	General function prediction only
S	191	6.5	Function unknown
-	1,049	28.7	Not in COGs

## References

[1] Wang Y Zhang Z Ruan J A proposal to transfer *Microbispora bispora* (Lechevalier 1965) to a new genus, *Thermobispora* gen. nov., as *Thermobispora bispora* comb. nov. Int J Syst Bacteriol 1996; 46:933-938 10.1099/00207713-46-4-933 8863419

[2] Henssen A Beiträge zur Morphologie und Systematik der thermophilen Actinomyceten. Arch Microbiol 1957; 26:373-414 10.1007/BF00407588 13435805

[3] Steger K Jarvis A Vasara T Romantschuk M Sundh I Effects of differing temperature management on development of *Actinobacteria* populations during composting. Res Microbiol 2007; 158:617-624 10.1016/j.resmic.2007.05.006 17683913

[4] Nonomura H, Ohara I. On a new actinomycete, *Microbispora*, isolated from soil. Abstract 98, pages 31 and 32, Abstracts of papers presented at the annual meeting of the Agricultural Chemical Society of Japan. Tokyo University, April 9th to llth, 1957. Published by the Agricultural Chemical Society (in Japanese). 1957.

[5] Henssen A Schnepf E Zur Kenntnis thermophiler Actinomyceten. Arch Microbiol 1967; 57:214-231 10.1007/BF00405948 5600778

[6] Lechevalier HA Priority of the generic name *Microbispora* over *Waksmania* and *Thermopolyspora.* Int Bull Bacteriol Nomencl Taxon 1965; 15:139-142 10.1099/00207713-15-3-139

[7] Miyadoh S Amano S Tohyama H Shomura T A taxonomic review of the genus *Microbispora* and a proposal to transfer two species to the genus *Actinomadura* and to combine ten species into *Microbispora rosea.* J Gen Microbiol 1990; 136:1905-1913 2283505 10.1099/00221287-136-9-1905

[8] Ochi K Haraguchi K Miyadoh S A taxonomic review of the genus *Microbispora* by analysis of ribosomal protein AT-L30. Int J Syst Bacteriol 1993; 43:58-62 10.1099/00207713-43-1-58 8427809

[9] Wang Y Zhang Z Ramanan N The actinomycete *Thermobispora bispora* contains two distinct types of transcriptionally active 16S rRNA genes. J Bacteriol 1997; 179:3270-3276 9150223 10.1128/jb.179.10.3270-3276.1997 PMC179106

[10] Chun J Lee JH Jung Y Kim M Kim S Kim BK Lim YW EzTaxon: a web-based tool for the identification of prokaryotes based on 16S ribosomal RNA gene sequences. Int J Syst Evol Microbiol 2007; 57:2259-2261 10.1099/ijs.0.64915-0 17911292

[11] Thawai C Tanasupawat S Kudo T *Micromonospora pattaloongensis* sp. nov., isolated from Thai mangrove forest. Int J Syst Evol Microbiol 2008; 58:1516-1521 10.1099/ijs.0.65410-0 18599686

[12] Tamura T Sakane T *Planotetraspora silvatica* sp. nov.,and emended description of the genus *Planotetraspora*. Int J Syst Evol Microbiol 2004; 54:2053-2056 10.1099/ijs.0.02981-0 15545433

[13] Garrity GM, Holt JG. The Road Map to the Manual. In: Garrity GM, Boone DR, Castenholz RW (eds), Bergey's Manual of Systematic Bacteriology, Second Edition, Volume 1, Springer, New York, 2001, p. 119-169.

[14] Zhi XY Li WJ Stackebrandt E An update of the structure and 16S rRNA gene sequence-based definition of higher ranks of the class *Actinobacteria*, with the proposal of two new suborders and four new families and emended descriptions of the existing higher taxa. Int J Syst Evol Microbiol 2009; 59:589-608 10.1099/ijs.0.65780-0 19244447

[15] Castresana J Selection of conserved blocks from multiple alignments for their use in phylogenetic analysis. Mol Biol Evol 2000; 17:540-552 10742046 10.1093/oxfordjournals.molbev.a026334

[16] Lee C Grasso C Sharlow MF Multiple sequence alignment using partial order graphs. Bioinformatics 2002; 18:452-464 10.1093/bioinformatics/18.3.452 11934745

[17] Stamatakis A Hoover P Rougemont J A rapid bootstrap algorithm for the RAxML web servers. Syst Biol 2008; 57:758-771 10.1080/10635150802429642 18853362

[18] Yarza P Richter M Peplies J Euzeby JP Amann R Schleifer KH Ludwig W Glöckner FO Rossello-Mora R The All-Species Living Tree project: A 16S rRNA-based phylogenetic tree of all sequenced type strains. Syst Appl Microbiol 2008; 31:241-250 10.1016/j.syapm.2008.07.001 18692976

[19] Liolios K Chen IM Mavromatis K Tavernarakis N Hugenholtz P Markowitz VM Kyrpides NC The Genomes On Line Database (GOLD) in 2009: status of genomic and metagenomic projects and their associated metadata. Nucleic Acids Res 2010; 38:D346-D354 10.1093/nar/gkp848 19914934 PMC2808860

[20] Nolan M Sikorski J Jando M Lucas S Lapidus A Glavina Del Rio T Chen F Tice H Pitlick S Cheng JF Complete genome sequence of *Streptosporangium roseum* type strain (NI 9100^T^). Stand Genomic Sci 2010; 2:29-37 10.4056/sigs.631049 PMC3035251 21304675

[21] Allgaier M Reddy A Park JI Ivanova N D'haeseleer P Lowry S Sapra R Hazen TC Simmons BA VanderGheynst JS et al. Targeted discovery of glycoside hydrolases from a switchgrass-adapted compost community. PLoS ONE 2010; 5:e8812 10.1371/journal.pone.0008812 20098679 PMC2809096

[22] Field D Garrity G Gray T Morrison N Selengut J Sterk P Tatusova T Thomson N Allen MJ Angiuoli SV The minimum information about a genome sequence (MIGS) specification. Nat Biotechnol 2008; 26:541-547 10.1038/nbt1360 18464787 PMC2409278

[23] Woese CR Kandler O Wheelis ML Towards a natural system of organisms: proposal for the domains *Archaea, Bacteria*, and Eucarya. Proc Natl Acad Sci USA 1990; 87:4576-4579 10.1073/pnas.87.12.4576 2112744 PMC54159

[24] Stackebrandt E Rainey FA Ward-Rainey NL Proposal for a new hierarchic classification system, *Actinobacteria* classis nov. Int J Syst Bacteriol 1997; 47:479-491 10.1099/00207713-47-2-479

[25] Classification of bacteria and archaea in risk groups. www.baua.de TRBA 466.

[26] Ashburner M Ball CA Blake JA Botstein D Butler H Cherry JM Davis AP Dolinski K Dwight SS Eppig JT Gene Ontology: tool for the unification of biology. Nat Genet 2000; 25:25-29 10.1038/75556 10802651 PMC3037419

[27] Klenk HP Göker M En route to a genome-based classification of *Archaea* and *Bacteria*?** Syst Appl Microbiol 2010; 33:175-182 10.1016/j.syapm.2010.03.003 20409658

[28] Wu D Hugenholtz P Mavromatis K Pukall R Dalin E Ivanova NN Kunin V Goodwin L Wu M Tindall BJ A phylogeny-driven genomic encyclopaedia of *Bacteria* and *Archaea*. Nature 2009; 462:1056-1060 10.1038/nature08656 20033048 PMC3073058

[29] List of growth media used at DSMZ: http://www.dsmz.de/microorganisms/media_list.php

[30] Sims D Brettin T Detter J Han C Lapidus A Copeland A Glavina Del Rio T Nolan M Chen F Lucas S Complete genome sequence of *Kytococcus sedentarius* type strain (541^T^). Stand Genomic Sci 2009; 1:12-20 10.4056/sigs.761 PMC3035214 21304632

[31] Hyatt D Chen GL LoCascio PF Land ML Larimer FW Hauser LJ Prodigal: prokaryotic gene recognition and translation initiation site identification. Bioinformatics 2010; 11:119 10.1186/1471-2105-11-119 20211023 PMC2848648

[32] Pati A Ivanova N Mikhailova N Ovchinikova G Hooper SD Lykidis A Kyrpides NC GenePRIMP: A gene prediction improvement ipeline for microbial genomes. Nat Methods 2010; 7:455-457 10.1038/nmeth.1457 20436475

[33] Markowitz VM Ivanova NN Chen IMA Chu K Kyrpides NC IMG ER: a system for microbial genome annotation expert review and curation. Bioinformatics 2009; 25:2271-2278 10.1093/bioinformatics/btp393 19561336

